# Clinical Evaluation of a Self-Testing Kit for Vaginal Infection Diagnosis

**DOI:** 10.1155/2021/4948954

**Published:** 2021-08-06

**Authors:** Ching-Ju Shen, Chung-Yao Yang, Huan-Yun Chen, Wei-Chun Chen, Ting-Chang Chang, Chao-Min Cheng

**Affiliations:** ^1^Department of Obstetrics and Gynecology, Kaohsiung Medical University Hospital and Pojen Hospital, Kaohsiung, Taiwan; ^2^Hygeia Touch Inc., Taipei, Taiwan; ^3^Department of Obstetrics and Gynecology, Kaohsiung Chang Gung Memorial Hospital, Kaohsiung, Taiwan; ^4^Department of Obstetrics and Gynecology, Chang Gung Memorial Hospital Linkou Branch, Taoyuan, Taiwan; ^5^Institute of Biomedical Engineering, National Tsing Hua University, Hsinchu, Taiwan

## Abstract

Vaginitis is a common disorder among women of varying ages that arises from a change in the normal pH balance of vaginal bacteria or an infection. Characteristic symptoms of itching, irritation, and odor cause considerable discomfort and increase the risk of contracting other sexually transmitted infections. Because of the sensitive and personal nature of the condition, some women may be reluctant to seek treatment. This behavior not only fails to solve the problem but may also delay medical treatment and result in additional medical complications. The pH changes associated with vaginitis and vaginosis, which are characterized by the presence or absence of inflammation, respectively, are well known but can vary. For example, bacterial vaginosis and trichomoniasis infection will raise vaginal pH above 4.5, while vulvovaginal candidiasis does not result in any measurable change to pH. Nonetheless, diagnostic tools relying on pH measurement are a valuable approach from which additional testing and treatment may be launched. Here, we focused on the use of a vaginal self-test tool and tested 50 patients, including pregnant women. When used according to the instructions, the Hygeia Touch Self-Testing Kit for Vaginal Infection demonstrated over 88% accuracy compared to a clinical diagnostic workup, with a sensitivity of 87% and a specificity of 89% in the patients where the swab was correctly interpreted. This study demonstrated an effective self-test method with high acceptability among women that provided them with greater autonomy regarding health management.

## 1. Introduction

Vaginal itching, pain, burning sensation, and abnormal vaginal discharge are common reasons for women to seek care in gynecological clinics, especially young women who have frequent sex and a higher incidence of vaginal infection compared to older women. Usually, these symptoms are related to abnormal vaginal flora (AVF), such as aerobic vaginitis (AV), bacterial vaginosis (BV), and trichomoniasis [1–4]. In the healthy vaginal environment, the most common bacteria belong to the *Lactobacillus* family, including strains such as *L. crispatus* and *L. gasseri*. *Lactobacillus* produces lactic acid, which is what protects the vagina against pathogens by stopping them from growing and prevents them from causing an infection that helps maintain a healthy microbial balance. Maintaining vaginal pH and microbial balance helps ward off invasive pathogenic fungi and protozoa. In general, a normal pH range for vaginal secretions is 3.8 to 4.5. Vaginal pH during vulvovaginal candidiasis infection is 4.0 to 4.7, usually <4.5, and a pH value higher than 4.5 may indicate BV or trichomoniasis [5, 6].

While physical examination provides useful diagnostic information, most studies have found that it is impossible to correctly diagnose the cause of vaginitis based solely on symptoms or the color of secretions, so laboratory diagnosis is necessary. Even experienced doctors find it difficult to correctly diagnose the cause of vaginitis, despite well-known and apparent symptomatology. This is further complicated by the fact that patients may have multiple, simultaneous infections, or they may be infected and not display or experience symptoms. Further, many clinics are not equipped for microscopic diagnosis. However, studies have shown that vaginal pH may be used as diagnostic criteria. Both BV and trichomoniasis are associated with vaginal pH levels greater than 4.5, but vulvovaginal *Candida* infection is associated with almost no change in vaginal pH [7–9]. While measuring vaginal pH is a useful screening method for diagnosing vaginal infections, it can be time-consuming during traditional gynecological examinations, and clinicians may struggle with consistent, standardized sample taking. Statistically, even trained physicians fail to make the correct diagnosis in approximately 40% to 50% of all patient examinations [10]. Currently, diagnosis by a general pharmacist is based only on a patient's oral description of their symptoms, which sometimes leads to the provision of incorrect, ineffective, and/or problematic prescriptions. For these reasons, an effective, rapid, and user-friendly tool to determine vaginal pH could prove to be highly useful.

Perhaps the most troublesome thing regarding vaginitis is recurrent infection. Since the US Food and Drug Administration (FDA) allowed the use of over-the-counter (OTC) vaginal antifungals in 1990 [11], the majority of women frequently make self-diagnosis and self-treatment via OTC pharmaceuticals instead of seeking medical treatment. This may be for a variety of reasons including convenience and the desire for rapid relief. As a result, reports of improper therapeutic approaches are frequent [12–15]. As a specific example, many patients experience dry, scum-like secretions without severe itching, and their self-diagnosis of *Candida* infection is unreliable, as more than half of them are incorrect. This condition requires clinical examination and diagnosis. Because the symptoms and manifestations of vaginal infections may be accompanied by mixed infections, it is difficult to correctly diagnose the infection cause. While it is good that more and more women pay attention to their health and seek self-care, many incorrectly self-medicate without seeking professional help. In addition, in many studies on self-collection of vaginal samples, no matter which method is used to test for vaginitis, most of them exclude pregnant women from self-collection [16, 17]. Providing a rapid, accurate, easy-to-use, and convenient vaginal pH screening tool would allow women (even pregnant women) to pursue more reliable self-care and allow clinicians to diagnose with greater confidence.

**Figure 1:**
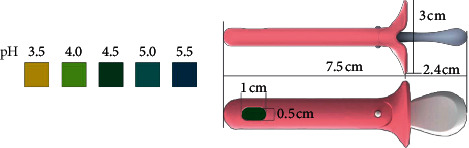
The OTC version of the pH self-test device is identical to the professional/prescription version, except for the package insert, which has been specifically modified to address the needs of the lay user. Both versions comprise a biocompatible grip with pH paper embedded in one end, a color chart, and a package insert. The device and color chart are illustrated above.

The Hygeia Touch Self-Testing Kit for Vaginal Infection (Hygeia Touch Inc., Taipei, Taiwan; MHW Medical Device Manufacturing, No. 006714) uses a vaginal applicator that includes a bromocresol green pH indicator embedded into a biocompatible grip (Figure 1). It was developed to detect vaginal pH, which can provide information regarding vaginal infections such as vulvovaginal candidiasis (VVC), BV, and trichomoniasis. If vaginal pH secretions are abnormal, that is, ≤4.5 or >4.7, the bromocresol green indicator may turn yellow or blue. Any slight changes in the color of the indicator should be considered useful for diagnostic considerations. According to the manufacturer, doctors can use this tool during consultations, but symptomatic patients may also use this tool themselves. This device has been registered with the US FDA.

The functional tip of this vaginal pH device is designed to accept pH test paper, which makes it more convenient for patients or doctors to obtain vaginal pH readings from the outer third of the vagina. This simplifies an otherwise difficult process and increases the likelihood of obtaining correct and useful readings. This study was conducted to determine whether patients can understand and use vaginal pH devices and to compare patient-determined results with results obtained by medical providers as a means of diagnosing vaginitis. The aim of this study was also to demonstrate that vaginitis can be correctly diagnosed through proper use and interpretation of vaginal pH devices that could then reduce the number of patients who abuse OTC antifungal drugs.

## 2. Subjects and Methods

The patients selected for this study were symptomatic, and the study was conducted in one private practice (Pojen Hospital) in Kaohsiung, Taiwan. The inclusion criteria for symptomatic women included vaginal symptoms or signs such as itching, burning, unpleasant odor, or abnormal discharge, and their menstrual cycles were normal. Patients were excluded if they could not read the instructions and/or were mentally or physically unable to perform the test. Patients were also excluded if they had douched or used contraceptive ointments or gels in the past 24 hours; had no protected sexual intercourse (using condoms) in the past 24 hours; and were currently menstruating or their last menstruation had ended less than 5 days prior. Women who were qualified for this study were asked to participate, and those who agreed signed informed consent (IRB No. 201900024A3).

In the examination room, physicians presented each patient with a vaginal pH device, instructions for use, and a subject questionnaire. Patients were then asked to read the instructions on their own before conducting the test in private. After the test was completed, each patient filled out their subject questionnaire and immediately handed their completed questionnaire back to the research nurse.

After patients completed their self-tests, the physician performed a physical examination in keeping with the nature of the patient's visit, including bacterial culture or microscopic examination, as needed. After the medical staff recorded the test results and completed the research questionnaire, they were instructed not to ask patients any other questions about the test. The test results are analyzed using standard statistical methods.

## 3. Results

This study recruited fifty subjects, all of which were symptomatic. The age of the subjects ranged from 19 to 68 years old (36 years on average, as shown in Table 1). Among them, 14 women were pregnant. This study primarily focused on patient self-care using the Hygeia Touch Self-Testing Kit for Vaginal Infection and the comparison of results to clinical diagnostic solutions based on the current gold standard diagnostic methods. In this study, the gold standard diagnostic method for vulvovaginal candidiasis was diagnosed by either a positive yeast culture or the presence of pseudohyphae or blastospores detected during microscopic examination. Trichomoniasis is using microscopy to confirm, while the diagnosis of BV is confirmed via Gram stain. Study sensitivity was defined as the percentage of patients who used the Hygeia Touch Self-Testing Kit for Vaginal Infection to self-determine vaginal pH at levels >4.5 (blue/green color), compared to the percentage of patients that received clinical diagnoses of BV/trichomoniasis. Specificity was defined as the percentage of patients who used the Hygeia Touch Self-Testing Kit for Vaginal Infection and self-diagnosed vaginal pH at levels ≤4.5 (green to yellow color change) that were clinically diagnosed with VVC or as normal. In other words, sensitivity is calculated based on how many people have BV or trichomoniasis. Specificity is calculated based on how many people do not have BV or trichomoniasis (e.g., normal or vulvovaginal candidiasis). Because it is recommended that patients see a doctor when the test results indicate BV or trichomoniasis (pH > 4.5), other test results (pH ≤ 4.5) would indicate that symptomatic patients should consider seeing a doctor to correctly diagnose abnormal conditions or that they should make self-treatment with OTC antifungal drugs. Therefore, we use pH = 4.5 as a cutoff value. Because there are currently no over-the-counter treatments for bacterial and trichomoniasis infections, the potential benefits of reducing the abuse of antifungal drugs in BV/trichomoniasis and normal cases clearly exceed the risk of false negative results. The physician's final diagnosis based on the results of the diagnostic gold standard was as follows: 11 subjects with BV, 23 with VVC, 3 with trichomoniasis, 1 with atrophic vaginitis, and 12 with no gross lesion (NGL). Vaginal pH test performance values are shown in Table 2. The sensitivity and specificity of the Hygeia Touch Self-Testing Kit for Vaginal Infection were 86.7% (13 of 15) (95% CI 69.5%–100%) and 88.6% (31 of 35) (95% CI 78.1%–99.1%), respectively. The accuracy was 88%. This study was deemed successful if the sensitivity of the Hygeia Touch Self-Testing Kit for Vaginal Infection was above 90% and if the specificity was above 70%. Diagnostic accuracy was also established by summing the number of correct assessments and dividing that number by the sample size.

The secondary outcome measure was the ease of use as judged by the patient who obtained the reading using the Hygeia Touch Self-Testing Kit for Vaginal Infection. Patients were given a questionnaire to complete after reading the package insert and performing the test. For this purpose, 8 questions and four-point response scales were used (see Table 3).

In this study, 50 patients were included and all of them returned the questionnaire. Regarding the kit design satisfaction question (Question 1), 100% answered “high” or “extremely.” Regarding the user manual readability question (Question 2), 100% responded with “high” or “extremely.” Regarding the willingness to use question (Question 3), 96% responded with “high” or “extremely.” Regarding the willingness to introduce the test to friends question (Question 4), 100% responded with “high” or “extremely.” Regarding the ease of use question (Question 5), 100% responded with “high” or “extremely.” Regarding the test safety question (Question 6), 100% responded with “high” or “extremely.” Regarding the kit comfort question (Question 7), 98% responded with “high” or “extremely.” Regarding the willingness to use question (Question 8), 98% responded with “high” or “extremely.” In a subgroup of pregnant women, 100% responded with “high” or “extremely” to all questions.

## 4. Discussion

This study shows that the design of the vaginal pH self-test device is suitable for home testing. The questionnaire survey found that almost all subjects thought the device was easy to use and the instructions were easy to read. The sensitivity, specificity, and accuracy of this study, which used the Hygeia Touch Self-Testing Kit for Vaginal Infection were 87%, 89%, and 88%, respectively. These results indicate that subjects could follow the instructions provided and make diagnostic conclusions in keeping with clinical diagnostics. Diagnostic differences and inaccuracies may be attributable to difficulties in distinguishing test paper color differences for pH 4.5 and pH 5.0, which were very similar.

Vaginitis is common in adult women and uncommon in prepubertal girls. Bacterial vaginosis accounts for 40–50% of vaginitis cases; vaginal candidiasis accounts for 20–25%; and trichomoniasis accounts for 15–20%. In US women of childbearing age, bacterial vaginosis is the most common vaginal infection. The test paper incorporated into the detection device used in this study produces color changes as the basis for diagnosis. In the presence of BV or *Trichomonas*, the color of this test paper remains unchanged or turns darker. In the presence of VVC, it turns yellow. In this study of Taiwanese patients, VVC was found in 46% (23/50) of those tested, while BV or trichomoniasis was found in 30% (15/50) of those tested. VVC is more prone to occur in Taiwan or subtropical countries because of the humid and hot weather. VVC was detected in 42.9% (6/14) of symptomatic pregnant women, while the prevalence rate of BV or trichomoniasis was 28.6% (4/14).

Traditionally, clinician diagnosis was based on clinical findings, physical examination, in-clinic tests, and medical history. However, accurate diagnosis is challenging due to infrequent use of office-based test which needs adequate tools and equipment [18]. Lack of access to point-of-care tools contributed clinician nonadherence to clinical practice guidelines in the diagnosis of vaginitis [19]. Sometimes, decisions are primarily based on a patient's description of symptoms, and sometimes existing symptoms may be complicated by the presence of other diseases. Both approaches may lead to misdiagnosis. The combination with Amsel criteria and Gram stain with Nugent scoring is recommended for the diagnosis of bacterial vaginosis [20] while the accuracy of Gram stain with Nugent scoring is strongly related to the reader's expertise. The components of criteria except for pH are either subjective or potentially time-consuming (use of a microscope to identify clue cells). Jane et al. reported pH had the highest sensitivity of all Amsel's test [21], and it can be easily performed in regular clinic setting.

Coinfection is common in vaginitis, making an accurate diagnosis and proper treatment challenging. Schwebke et al. reported coinfection rates by 2 or more organisms were 20% by reference testing and approximately 25% by investigational testing [22]. According to numerous literature about mixed infection, when the clinician's diagnosis based on physical examination and clinical symptoms is different from the self-test kit, the possibility of coinfection should be considered. Physiological vaginal discharge usually does not require treatment, but sometimes when the discharge is abnormal or when there is discomfort such as itching, burning, or pain, many women think that it is caused by VVC. However, Ferris et al. found that only 33.7% of people using OTC fungal self-treatment found effective relief [14]. This study also notes that most women who think they have VVC actually have BV. Improper use of OTC antifungal drugs disrupts the normal vaginal environment, leading to more serious complications and infections, especially for patients that have a BV infection [15, 23–29].

Currently, there are a few commercially available medical devices for self-diagnosis of vaginitis. Most of them are designed to change color when detecting BV, and the test paper or cotton swab element in them change from yellow to green to indicate a positive result [26]. This study also summarizes and compares the vaginitis self-diagnostic tools currently available on the market (Table 4). From a design perspective, the Hygeia Touch Self-Testing Kit for Vaginal Infection is optimized for product safety (Patent No. US D869,679S). The double-layer structure ensures that the chemical substance on the test paper does not directly touch the vaginal mucosa, and it also ensures that the test paper does not fall from the device. The design of the petal stopper can also prevent the user from inserting the sampling device too deeply into the vagina. In terms of comfort, most subjects can accept self-diagnostic tools, so they are relatively incomparable.

Because it is recommended that patients see a doctor when the test results indicate BV or trichomoniasis (pH > 4.5), other test results (pH ≤ 4.5) would indicate that symptomatic patients should consider seeing a doctor to correctly diagnose abnormal conditions or that they should make self-treatment with OTC antifungal drugs. Because there are currently no over-the-counter treatments for bacterial and trichomoniasis infections, the potential benefits of reducing the abuse of antifungal drugs in BV/trichomoniasis and normal cases clearly exceed the risk of false negative results. In the case of increased costs for publicly funded health systems, the accuracy of self-diagnosis and treatment should be encouraged. In addition, because vaginal swabs will only lead to correct measures when the results are considered based on the patient's symptoms, it is possible for pharmacists to assist patients in self-care. When symptoms occur and the Hygeia Touch Self-Testing Kit for Vaginal Infection indicates a vaginal pH ≤ 4.5, the result can still be used by healthcare providers or patients themselves and is relevant for treatment decisions. This test helps to provide information on BV/trichomoniasis and VVC by determining the change in vaginal pH. Also, because BV or trichomoniasis are relatively serious symptoms, in terms of medical testing, it is aimed that the false negative rate can be as low as possible. In this study, the false negative rate of using Hygeia Touch Self-Testing Kit for Vaginal Infection is about 6.1%. This is a simple, single-step test that does not require complicated procedures for interpretation and can be read immediately.

It is worth noting that vaginal pH self-test devices have shown sufficient evidence in other literature [7–9]. This supports the idea that vaginal pH self-test device performance is reasonable, acceptable, and helpful in the diagnosis of vaginitis. Furthermore, in response to the COVID-19 pandemic, the demand for telemedicine is dramatically increasing to limit physical contact. Evidence-based guidance for telemedicine in gynecological conditions has been made to assist health providers in delivering adequate and safe healthcare [30]. However, key limitations of this “touchless care” are the lack of comprehensive physical examination and immediate specimen collection. Combining a diagnostic tool which offers ease of use and clear result with patient reported symptoms permits more accurate diagnosis in telehealth services. Proper use of such devices can lead to the more appropriate use of OTC antifungal drugs and encourage healthcare providers to appropriately use such devices to assist in diagnosis and improve women's health.

## 5. Conclusions

The self-test device and approach described here exhibited high acceptability among women and provided them with greater autonomy in regard to health management. It was also an accurate and observable method for diagnosing vaginal infection, which may cause adverse outcomes including increased risk of pelvic inflammation disease and infertility. Because pregnant women have been shown to be more likely than nonpregnant women to experience asymptomatic *Candida*-associated vulvovaginal infection and this tool demonstrated a high satisfaction level when used as a self-test tool, we believe it could be highly suitable for genital tract health assessment during pregnancy. Although the pH sensing was used in traditional chemical methods, with safe self-collection tools, sensors with more detection mechanisms (i.e., electrochemical) can be integrated in the future and applied to other vaginal environments measurement, such as strain analysis. In summary, this vaginitis self-diagnostic tool is a reliable and effective device suitable for home-based care and clinical, point-of-care testing.

## Figures and Tables

**Table 1:** Clinical characteristics of enrolled patients.

Clinical characteristics	Value
*Age, years*	
Average (*n* = 50)	36
Maximum	68
Minimum	19

*Pregnant or not*	
Average (*n* = 50)	50
Pregnant	14
Not pregnant	36

**Table 2:** Patient pH readings versus healthcare providers' diagnoses.

Patients' initial self pH readings	Healthcare providers' diagnoses		
	Bacteria (*Trichomonas*)	Yeast (candidiasis) or normal	Total
pH > 4.5	13	4	17
pH ≤ 4.5	2	31	33
Total	15	35	50
Sensitivity (%)	86.7		
Specificity (%)		88.6	
Accuracy (%)			88

**Table 3:** Questions regarding the comfort of use.

Question 1	The satisfaction level of the design of the kit
Question 2	How easy is it for you to read the user manual?
Question 3	The willingness to use this kit as prescreening while vaginal symptom occurs?
Question 4	The willingness to introduce the kit to your friends?
Question 5	The ease level to put the kit into the vagina and pull it out and check the test result?
Question 6	Do you feel it is a safe kit after using it?
Question 7	Do you feel comfortable after using the kit?
Question 8	The willingness to use this kit as prescreening while vaginal symptom occurs after you use it this time?
The possible responses were as follows: not at all, low, high, extremely	

**Table 4:** Summary of vaginitis self-diagnostic devices on the market.

	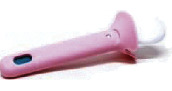	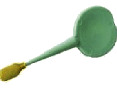		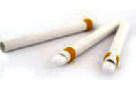	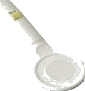
Brand	Hygeia Touch	Monistat	Walgreen, rite-aid, CVS	EcoCare comfort	Biosynex
Product appeal	Vaginal infections, pregnant preparation	Vaginal infections	Vaginal infections	Pregnant, menopause	Vaginal infections
Accuracy (%)^*∗*^	88	92		—	90
Test/pack	1	2	2	10	3
Differentiation	The double-layer structure is optimized for the safety of the product	Treatment bundled	—	—	—

^*∗*^The accuracy of Monistat and Biosynex is according to the product user manuals.

## Data Availability

All data have been included within the manuscript. If necessary, all data are also available on request through direct contact with the corresponding author.
